# The HPV E2 Transcriptional Transactivation Protein Stimulates Cellular DNA Polymerase Epsilon

**DOI:** 10.3390/v10060321

**Published:** 2018-06-12

**Authors:** Michaelle Chojnacki, Thomas Melendy

**Affiliations:** Departments of Microbiology & Immunology and Biochemistry, Jacobs School of Medicine & Biomedical Sciences, University at Buffalo, Buffalo, NY 14214, USA; mdc37@buffalo.edu

**Keywords:** human papillomavirus, DNA replication, DNA polymerase epsilon

## Abstract

The papillomavirus (PV) protein E2 is one of only two proteins required for viral DNA replication. E2 is the viral transcriptional regulator/activation protein as well as the initiator of viral DNA replication. E2 is known to interact with various cellular DNA replication proteins, including the PV E1 protein, the cellular ssDNA binding complex (RPA), and topoisomerase I. Recently, we observed that cellular DNA polymerase ε (pol ε) interacts with the PV helicase protein, E1. E1 stimulates its activity with a very high degree of specificity, implicating pol ε in PV DNA replication. In this paper, we evaluated whether E2 also shows a functional interaction with pol ε. We found that E2 stimulates the DNA synthesis activity of pol ε, independently of pol ε’ s processivity factors, RFC, PCNA, and RPA, or E1. This appears to be specific for pol ε, as cellular DNA polymerase δ is unaffected by E1. However, unlike other known stimulatory factors of pol ε, E2 does not affect the processivity of pol ε. The domains of E2 were analyzed individually and in combination for their ability to stimulate pol ε. Both the transactivation and hinge domains were found to be important for this stimulation, while the E2 DNA-binding domain was dispensable. These findings support a role for E2 beyond E1 recruitment in viral DNA replication, demonstrate a novel functional interaction in PV DNA replication, and further implicate cellular pol ε in PV DNA replication.

## 1. Introduction

Papillomaviruses (PV) encode only two proteins required for viral DNA replication: the viral hexameric DNA helicase, E1, and the multifunctional transcriptional regulator and replication initiator protein, E2 [1,2]. E2 is required for recruiting and loading E1 onto the viral origin of replication [3,4,5]. Although E1 is the primary viral DNA replication protein, E2 plays an important secondary role in helping E1 bind to the origin and assemble into hexamers [6]. Other than E1 and E2, PV DNA replication is carried out completely by host DNA replication machinery [7].

E2 has three distinct domains, each of which can be separated by function. The amino-terminal 200 amino acids comprise the transactivation domain. Residues within the transactivation domain (TAD) are important for both transcriptional activation and repression as well as DNA replication [8]. Residues within this domain are also important for interaction with E1 [8,9,10,11]. The C-terminal domain is approximately 85–100 amino acids and is known as the DNA-binding domain (DBD) [12]. The DBD is important for sequence recognition and is also important for forming stable E2 dimers [13]. E2 also contains a hinge region (H) that is not well conserved and is variable in both sequence composition and length [12,14]. The E2 hinge is thought to be an unstructured link between the transactivation and DNA-binding domains [8]. Nevertheless, the hinge region is important for nuclear localization, binding to chromatin, and interacts with many cellular proteins such as histone acetyltransferases, splicing factors, and transcription factors, among others [8,15,16]. Deletions of the hinge region were found to be defective for viral DNA replication [17].

E2 has been shown to interact with or modulate the activity of several cellular proteins. These include RPA, topoisomerase I (topo I), and p53, among others [8,18,19]. Of possible relevance to HPV DNA replication, E2 was observed to interact with and stimulate the relaxation activity of topo I [18]. Because the ability of topo I to relieve torsional strain on DNA is essential for DNA replication, these findings suggest that the E2-topo I interaction may be an important part of the DNA replication process, possibly playing a role in the elongation stage of PV DNA replication.

We recently reported that HPV E1 helicase binds to, stimulates, and confers processivity to pol ε [20]. These findings, as well as previous findings that E2 modulates activities of other cellular replication proteins, led us to evaluate whether E2 modulates the activity of pol ε.

## 2. Materials and Methods

### 2.1. Purification of Recombinant Proteins

HPV 11 glutathione S-transferase (GST)-E2 truncated constructs were created using the Takara In-Fusion cloning kit (Takara Bio USA, Inc., Mountain View, CA, USA). HPV 11 E2 transactivation domain consists of amino acids 1–202. HPV 11 E2 hinge domain consists of amino acids 203–286. HPV 11 E2 DNA-binding domain consists of amino acids 287–365. We PCR amplified these regions of the E2 ORF individually and pair-wise and integrated them into pGex6p as per the manufacturer’s instructions. Construct cloning was verified by DNA sequencing. The HPV 11 maltose binding protein (MBP)-TAD-H construct was also created using the Takara In-Fusion cloning kit, in a similar manner to the creation of the GST-E2 proteins, but into the pMALC2 expression vector (New England Biolabs, Inc., Ipswich, MA, USA), rather than into pGex6p. All clones were verified by DNA sequencing.

GST-E2 proteins were purified from *Escherichia coli* using GST-affinity. Briefly, the various GST-E2 constructs were transformed into *E. coli* strain BL21 (DE3). The cultures were grown to A_595_ = 0.4 at 37 °C, the temperature reduced to 18 °C, further grown to A_595_ = 0.6, and isopropyl β-d-1-thiogalactopyranoside (IPTG) was added to 0.3 mM to induce expression. After 16 h of induction at 18 °C, cells were collected by centrifugation (5000× *g* for 20 min at 4 °C), washed with cold phosphate buffered saline (PBS) and again subjected to centrifugation (5000× *g* for 20 min at 4 °C). Cells were re-suspended in 25 mL of ice cold Buffer A (50 mM Tris-HCl (pH 7.5), 100 mM NaCl, 10 mM EDTA, 2 mM DTT, 20% sucrose and 1 mM PMSF). The cell suspensions were subjected to 1500 pounds per square inch for 5 min in a 4 °C Parr^TM^ cell disruption chamber, and the suspension released slowly to lyse. Lysates were then sonicated for five 20 s pulses on ice. NP40 was added to 0.1% final concentration and the lysates were subjected to centrifugation for 20 min at 20,000× *g* at 4 °C. One mL of pre-equilibrated glutathione Sepharose (GE Healthcare Life Sciences, Chicago, IL, USA) was added to the lysates and incubated for three hours at 4 °C. The resin was washed twice with 40 mL of Buffer A and poured into column format. The resin was washed with 5 mL Buffer A containing 0.1 mM PMSF, 5 mL of Buffer B (50 mM Tris-HCl (pH 8.8), 1 mM EDTA, 2 mM DTT, 10% glycerol) with 1 M NaCl, and then 5 mL of Buffer B with 0.2 M NaCl. Protein was then eluted with 6 mL of Buffer B containing 0.2 M NaCl and 10 mM glutathione. Fractions were evaluated using SDS-PAGE and fractions containing the GST-E2 construct were pooled.

The MBP-TAD-H protein was purified from *E. coli* using maltose binding protein (MBP) affinity. Briefly, transformed BL21 (DE3) *E. coli* was grown to A_595_ = 0.4 at 37 °C. The temperature was reduced to 18 °C, further grown to A_595_ = 0.6, and isopropyl β-d-1-thiogalactopyranoside (IPTG) was added to 0.4 mM to induce expression. Cells were grown for 2 h at 18 °C. Cells were collected by centrifugation (5000× *g* for 20 min at 4 °C), washed with cold PBS, and again collected by centrifugation (5000× *g* for 20 min at 4 °C). Cells were frozen using liquid nitrogen and stored at −80 °C. Before use, cells were thawed on ice and resuspended in 25 mL MBP column buffer (20 mM Tris-HCl (pH 7.5), 200 mM NaCl, 1 mM EDTA, 1 mM DTT, 1 mM PMSF, 5% glycerol, 0.01% NP40). The cell suspension was subjected to 1500 psi for 5 min in a cold Parr^TM^ cell disruption chamber, and the suspension released slowly to lyse. Lysates were then sonicated for five 20 s pulses on ice and subjected to centrifugation for 20 min at 20,000× *g* at 4 °C. Supernatants were collected, diluted 1:6 in MBP column buffer, and applied to 1 mL of pre-equilibrated amylose resin (New England Biolabs, Inc., Ipswich, MA, USA) Resin was washed with 20 mL MBP column buffer containing 1 M NaCl, and further washed with 20 mL MBP column buffer containing 0.2 M NaCl. MBP-TAD-H was eluted using 2 mL MBP column buffer containing 10 mM maltose. Fractions were evaluated using SDS-PAGE and protein peak fractions were pooled.

HPV 11 EE-E1 was expressed in High Five insect cells using baculovirus expression as described [20]. Ten T150 flasks of High Five insect cells were infected at a multiplicity of infection of 3 for 1 h at 27 °C. After 48 h, cells were harvested and washed with PBS. Twenty mL of ice cold lysis buffer (20 mM Hepes-NaOH (pH 7.5), 400 mM NaCl, 0.5 mM EDTA, 1 mM MgCl_2_, 1 mM DTT, 1 mM PMSF) was added to the cell pellet, which was then resuspended, incubated on ice for 10 min, and then subjected to Dounce homogenization by 20 strokes. Lysates were subjected to centrifugation (31,000× *g*) for 10 min at 4 °C. The supernatant was diluted with Buffer Q (20 mM Tris-HCl, (pH 7.0) and 10 mM 2-mercaptoethanol) until the ionic strength was equivalent to Buffer Q with 50 mM NaCl. This was applied to a 10 mL Q Sepharose column. After washing with 50 mL Buffer Q containing 50 mM NaCl, the column was eluted with 10 mL Buffer Q containing 500 mM NaCl. The peak protein-containing fractions were pooled and applied to a 1 mL affinity column (anti-EE monoclonal antibody conjugated to Protein A Sepharose 4B; [21]). After extensive washing with 20 mM Tris-HCl (pH 7.0) with 0.5 M NaCl, the EE-E1 was eluted with 5 mL of 50 mM triethylamine (pH 11.5). Half mL fractions were collected into 50 μL Tris-HCl (1.0 M pH 7.0) to neutralize. SDS-PAGE was used to analyze the fractions for EE-E1. EE-E1 eluates were concentrated using Millipore Centricon (10 kDa MWCO) (Burlington, MA, USA).

DNA polymerases δ and ε were prepared as full complexes using multiple baculovirus infection expression and purified as described [22]. Recombinant bacterially expressed human RPA 3-subunit complex was prepared as described [23]. PCNA was over-expressed in *E. coli* and purified as described [24]. The human RFC full 5-subunit complex was expressed using a five baculovirus infection expression and purified as described [25].

### 2.2. Primer Extension Assays

The primed M13 polymerase assays was carried out at described [20]. Briefly, M13 ssDNA was annealed to a 17 nucleotide oligonucleotide (5′-GTAAAACGACGGCCAGT-3′, 1 pmol) at an approximate 1:1 ratio. Polymerase reactions were performed in 10 μL reaction volumes with 100 ng of primed M13 (except as indicated for processivity assays), 0.1 mg/mL acetylated BSA, DNA replication buffer (40 mM sodium creatine phosphate, 20 mM Tris-HCl (pH 7.5), 7 mM MgCl_2_, 4 mM ATP, 200 μM each of CTP, UTP and GTP, 25 μM dATP, 100 μM each of dCTP, dGTP and dTTP and 0.5 mM DTT), and ^32^P-dATP (~3000 cpm/pmol, 1 μCi per reaction). The replication factors RPA, RFC, PCNA, pol ε, pol δ, HPV 11 EE-E1, and HPV 11 GST-E2 were individually titrated into the reactions to establish optimal DNA synthesis activity and were used at the following concentrations: RFC (3 ng/μL), PCNA (10 ng/μL), RPA (70 ng/μL), Pol ε (1 ng/μL unless otherwise indicated), HPV E1 (60 ng/μL unless indicated otherwise), HPV 11 E2 (20 ng/μL unless indicated otherwise). After incubation at 37 °C for 1.5 h (unless indicated otherwise), reactions were terminated (with 2% SDS (wt/vol), 50 mM EDTA, 5% glycerol (vol/vol) and 0.2 mg/mL proteinase K for 15 min at 37 °C), and DNA products were then denatured (by addition of 0.25 M NaOH, 2.5 mM EDTA, 2.5% Ficoll (*w*/*v*))and subjected to electrophoretic separation on a 25 cm × 20 cm 1% agarose in 50 mM NaOH and 1 mM Na_2_EDTA at 40 V for 16 h at room temperature Gels were fixed and subjected to phosphorimager analysis. Quantifications were performed using ImageJ software (National Institute of Health, Bethesda, MD, USA). All replicates of each experiment were quantified by boxing the entire lane, and a background (no sample lane) was subtracted from the experimental values. All experiments were performed at least three independent times. Experiments were performed with polymerases prepared using baculovirus expression both in our own laboratory as well as with polymerase samples prepared in the laboratory of Dr. Jerard Hurwitz (Memorial Sloan Kettering); similar results were obtained with preparations from both sources. As a result of isotope variation, quantifications across multiple experiments were plotted as percent synthesis, with pol ε in the presence of replication factors (RFC, PCNA, and RPA) set to 100% as an internal standard for each experiment.

The molecular weight marker was created by end-labeling the GeneRuler 1 kb ladder (Thermofisher Scientific, Waltham, MA, USA) with γ-^32^P-ATP (3000 cpm/pmol) using T4 polynucleotide kinase as per instructions (Thermo Scientific). A second “marker” was presented in each gel created by carrying out a polymerase reaction containing pol ε and Replication Factors (RF) (as described above) to create a standard spectrum “smear”. Precise molecular weight/banding was not required for our analyses/quantification but, to provide information on relative strand length, these markers were run on a parallel gel run identically to the reaction gels and were only present to demonstrate approximate strand length.

### 2.3. Statistics

Unpaired Student’s *t* tests were performed using GraphPad software (GraphPad, San Diego, CA, USA) Analyses were conducted with a 95% confidence interval.

## 3. Results

### 3.1. HPV E2 Stimulates Pol ε But Not Pol δ

**Figure 1:**
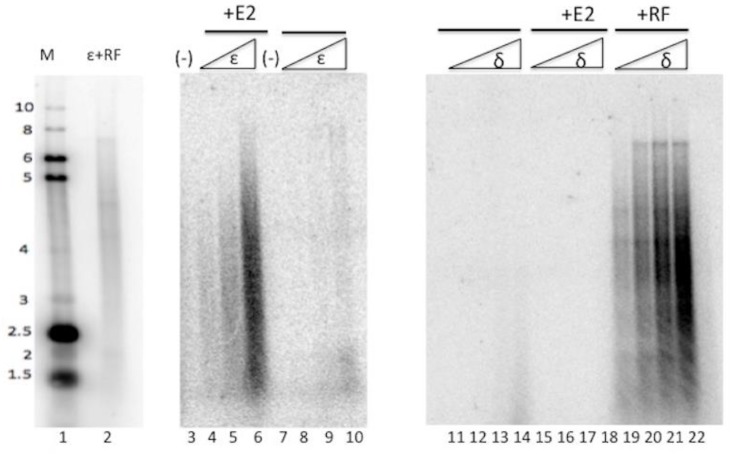
HPV E2 stimulates pol ε. Pol ε was added to primed M13 reactions containing 40 ng/μL of HPV 11 GST-E2 (lanes 2–4) or to reactions without E2 (lanes 6–8). Pol ε was added in amounts ranging from 1 ng/μL to 8 ng/μL (lanes 4–6 and 8–10). Pol δ was added in increasing amounts (1 ng/μL to 8 ng/μL) to reactions containing E2 (lanes 15–18) or replication factors (RF, as described in Methods) (lanes 19–22). Lanes 11–14 represents pol δ alone. Lanes 3 and 7 represent control lanes in which no pol ε was present in the reactions. Lane 1 shows reference markers (in kb) run on a parallel gel to provide size of nascent strands, and lane 2 shows the product spectrum generated by pol ε and its cellular cofactors, as described in Materials and Methods.

**Figure 2:**
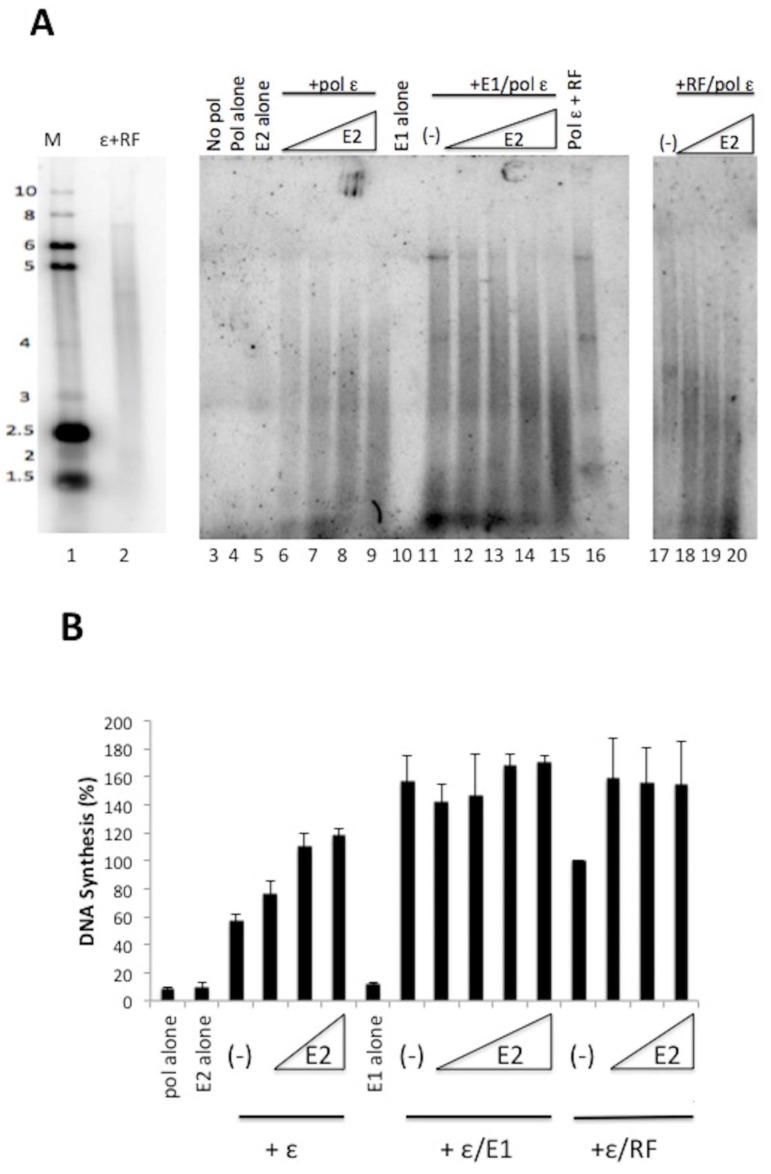
HPV E2 does not act synergistically with HPV E1 or RF to stimulate pol ε. (**A**) Primed M13 assays were used to evaluate the combined effect of E2 with E1 or RF on pol ε. HPV 11 GST-E2 was added in increasing amounts (20 ng/μL to 200 ng/μL) to reactions containing pol ε (lanes 6–9), pol ε with 60 ng/μL of HPV E1 (lanes 12–15), and pol ε with RF (lanes 18–20). Lanes 3–5 and 10 represent controls. Lanes 11, 16, and 17 represent the stimulation of pol ε by either E1 or RF in the absence of E2. Lanes 1 and 2 show the same gel size markers as described in Figure 1. (**B**) Quantification of results of (**A**) and repeat experiments using phosphorimage analysis as described in the Methods. Pol ε + RF was set to 100% and all other values were plotted relative to this. Experiments were done three times and standard deviations were based on three experiments. An unpaired Student’s *t* test (GraphPad software) was used to compare the effect of E2 on pol ε with the combined effect of E2 and RF on pol ε. The *p* value was 0.26 and not found to be statistically significant.

PV E2 has interactions with various cellular proteins, including functional interactions with topo I [18,19,26]. Because we observed modulation of pol ε’ s activity by HPV E1, we analyzed whether HPV E2 would affect the activity of pol ε. Primed M13 assays were employed to determine if E2 would either stimulate or inhibit the DNA synthesis activity of pol ε (Figure 1). Pol ε was added in increasing amounts to a constant amount of E2 and it was observed that E2 stimulated the DNA synthesis activity of pol ε; this was not due to a non-specific protein stabilization effect, as the same levels of pol ε added to reactions with saturating protein stabilizing levels of acetylated bovine serum albumin showed dramatically lower levels of pol ε activity (compare lanes 4–6 to lanes 8–10). This was a highly specific stimulation, as DNA polymerase delta (pol δ), which is the DNA polymerase most closely related to pol ε both evolutionarily and functionally [27], showed no stimulation by E2 (Figure 1, compare lanes 15–18 to lanes 11–14). That our pol δ preparation was functional and capable of being stimulated by its known co-factors, was demonstrated by pol δ being highly stimulated by RFC, PCNA, and RPA (Figure 1, RF, lanes 19–22). We also demonstrated below that pol ε was appropriately stimulated by RFC, PCNA, and RPA, as seen in Figure 2. (Note that the banding pattern sometimes detected in these DNA polymerase assay products are a result of the single-strand DNA sequence of M13 that produces a great degree of secondary structure; different polymerase preparations and co-factor additions frequently result in variable banding patterns. Other than overall appearance of very long versus short products, the specific patterns are essentially inconsequential. We have never detected differences in stimulation levels of pol ε due to RF or E1 in reactions with differing banding patterns in these M13 assays.) The results in Figure 1 demonstrate that pol ε was stimulated specifically by E2, and that this stimulation was specific for DNA polymerase ε and not seen with DNA polymerase δ.

### 3.2. HPV E2 Does Not Act Synergistically with HPV E1 or Replication Factors to Stimulate Pol ε

We observed that HPV E2 stimulated the DNA synthesis activity of pol ε in the absence of pol ε’s processivity cofactors RFC, PCNA, and RPA (Figure 1). To evaluate whether E2 could have additive effects with other pol ε stimulatory factors, we analyzed the effect of E2 on pol ε in the presence of either E1 or RF (Figure 2). When pol ε was in the presence of increasing amounts of E2, we observed stimulation of DNA synthesis by pol ε (Figure 2A, lanes 6–9). We analyzed the combined effect of E1 and E2 on pol ε with the addition of a constant amount of pol ε (1 ng/μL) and E1 (60 ng/μL, lanes 11–15), which we have shown to have stimulatory effects on pol ε [20] and increasing amounts of E2. E1 stimulates pol ε to synthesize longer chains of DNA (lane 11). With the addition of E2 (lanes 12–15), we observed the shift from long DNA products to short DNA products as the concentration of E2 increased. (This decrease in nascent strand length was sometimes also seen with pol ε and E2 alone (Figure 2A, lanes 6–9)). Thus, in the presence of both E1 and E2, we did not observe an additive stimulation of pol ε but rather an apparent competition of one stimulatory factor for another (Figure 2B, also note the similarity in product size in lanes 15 and 9 of Figure 2, Panel A). We also combined E2 and RF (lanes 17–20). Again, as E2 concentrations increased DNA product length decreased, resulting in products closer in size to those seen with pol ε and E2 alone, which suggests a competition between the two types of stimulatory factors. Unlike with E1, E2 stimulated pol ε up to an additional 50% in the presence of RF, indicating that stimulatory effects of RF and E2 are partially additive; however, this was not found to be statistically significant, as determined by the Student’s *t* test (*p* = 0.26).

### 3.3. E2 Does Not Confer Processivity to Pol ε

**Figure 3:**
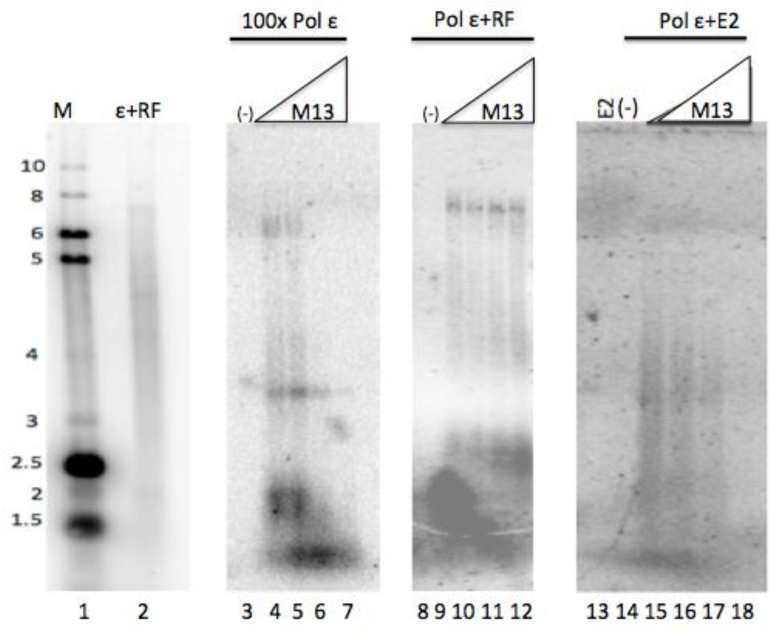
E2 does not confer processivity to pol ε. Pol ε processivity was examined by performing primed M13 reactions under conditions in which template was in vast excess of polymerase [28,29]. Pol ε (0.1 ng/μL, 2.9 fmoles) was incubated with E2 (40 ng/μL) with increasing concentrations of template (5–40 fmoles, lanes 15–18) or with RF (as described in Methods) with increasing concentrations of template (lanes 9–12). A high concentration of pol ε (10 ng/μL) was similarly incubated with increasing concentrations of the primed M13 template (lanes 4–7). No template was present in lanes 3, 8, and 14. Lanes 1 and 2 show the same gel size markers as described in Figure 1.

Other factors that stimulate pol ε, such as RF (RFC, PCNA, and RPA) as well as HPV E1, increase pol ε’s processivity and its ability to synthesize long DNA products. Because E2 was observed to stimulate pol ε, we evaluated whether E2 could confer processivity to pol ε (Figure 3). Single-primed ssM13 template was added in increasing concentrations so that the template was in great excess of pol ε [28,29]. Under these conditions, the degree of extension of the primer is indicative of the polymerase’s processivity; if additional templates do not result in shorter products, then the polymerase must remain associated with the initial template it bound to. When pol ε was combined with RF, long DNA product lengths (up to full-length, ~7 kb) were observed under all concentrations of template, indicating pol ε synthesis in the presence of RF is processive (lanes 9–12). Conversely, synthesis by pol ε without any co-factors showed a dramatic decrease in nascent strand length and overall inhibition with increasing levels of template, indicative of non-processive DNA synthesis (note that 100-fold higher levels of pol ε had to be used to obtain detectable levels of synthesis, lanes 4–7). The DNA products synthesized in the presence of E2 were intermediate in length between those synthesized by pol ε alone and those synthesized by pol ε with RF (Figure 2, lane 15 compared to lanes 4–7 and lanes 9–12). Addition of increasing amounts of template resulted in a shortening of the product length by stimulation of pol ε in the presence of E2 as well as an overall inhibition of synthesis in the reaction. These results are consistent with E2-stimulated synthesis of pol ε being non-processive. Hence, the stimulation of pol ε by E2 appears to not be due to an increase in processivity.

### 3.4. The E2 Transactivation and Hinge Domains Are Important for Stimulation of Pol ε

**Figure 4:**
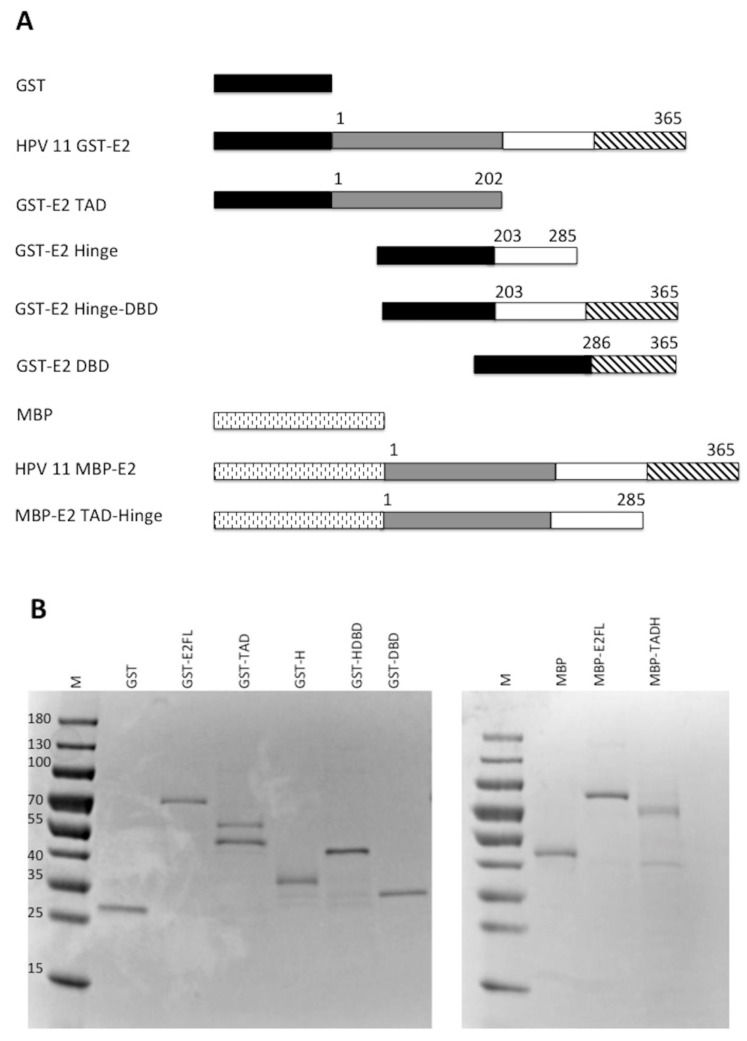

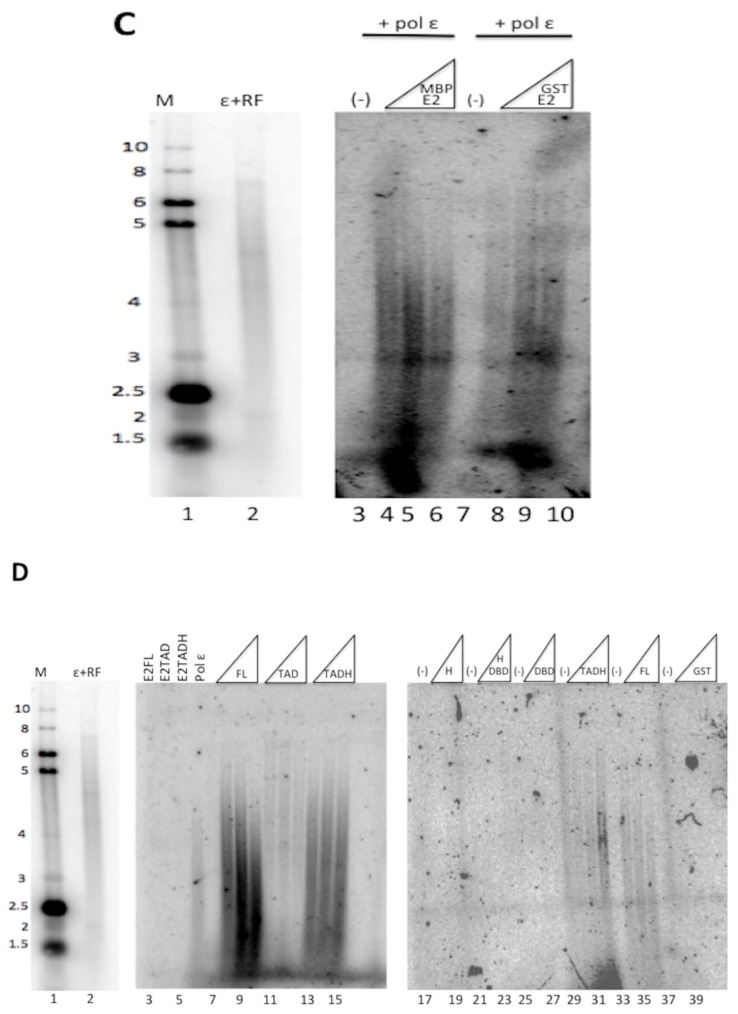

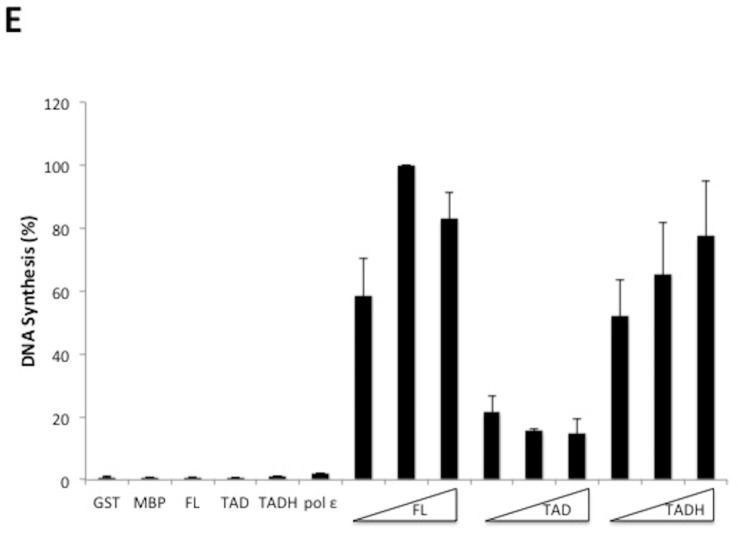
The TAD and hinge regions are important for stimulation of pol ε. (**A**) Various HPV 11 GST-E2 domain fusion proteins with MBP and GST were constructed and purified as described in the Methods. GST is approximately 200 amino acids, MBP is approximately 380 amino acids, and the orientation and relative size of each of the fusion proteins is pictorially represented. (**B**) 0.5 μg of each purified fusion protein was separated by SDS-PAGE and stained with Coomassie. (**C**) M13 primer extension assays were performed using 1 ng/μL of pol ε per reaction and 5, 10 and 20 pmoles of either MBP-E2 (lanes 4–6) or GST-E2 (lanes 8–10). Lanes 1 and 2 show the same gel size markers as described in Figure 1. (**D**) M13 primer extension assays were performed using 1 ng/μL of pol ε per reaction and 5, 10, and 20 pmoles of recombinant tagged E2 construct or protein tag. Lanes 8–10 and 34–36 represent the GST-tagged full-length protein (FL), lanes 11–13 represent the GST-tagged transactivation domain (TAD), lanes 14–16 and 29–32 represent MBP-tagged transactivation-hinge domains (TADH), lanes 15–18 represent GST-tagged hinge (H), lanes 21–24 represent the GST-tagged hinge-DNA-binding domain (HDBD) and lanes 25–28 represent the GST-tagged DNA-binding domain (DBD). GST alone was also purified and evaluated (lanes 37–40) as was MBP alone (**E**). Lanes 3–5 and 7,17, 21, 25, 29, 33, and 37 contain 20 pmoles of the purified E2 construct designated without pol ε. Every other lane was annotated as a result of space constraints. Lanes 1 and 2 show the same gel size markers as described in Figure 1. (**E**) Quantification of results of (**C**) and repeat experiments using phosphorimage analysis as described in the Methods. Pol ε + E2 (10 pmoles) was set to 100% for each experiment, and all other values were quantified relative to the 100% control for that set of reactions. Experiments were performed three times and standard deviations were based on three experiments, with the exception of the MBP tag, which was only done once. An unpaired Student’s *t* test was performed (GraphPad software) to compare the effect of each of the FL, TAD, and TADH constructs on pol ε at a 95% confidence interval. Stimulation of pol ε by full-length E2 compared to TAD was found to be significant (*p* < 0.0001) and TAD compared to the control lanes was also found to be significant (*p* = 0.0001). The difference in stimulation of pol ε between TAD and TADH was also found to be statistically significant (*p* < 0.0001). Full length was also compared to TADH and was not found to be statistically significant (*p* = 0.1710).

E2 is comprised of three distinct domains: the transactivation domain (TAD), the DNA binding domain (DBD), and the flexible hinge domain (H) that connects the other two domains [8,30]. Through truncating E2, it was previously observed that the transcriptional activation and replication functions could be separated [17]. To evaluate which domains of E2 were important for the stimulation of pol ε, glutathione S-transferase (GST) and maltose binding protein (MBP) constructs were created that expressed each domain of HPV11 E2, DBD, TAD, and H, both alone and in combination with their adjacent domain(s) (Figure 4A). These were used to express the protein constructs that were then purified for biochemical analyses (Figure 4B). Degradation products were detected for some of the constructs but, in most cases, were not observed to be the major protein purified. The GST-TAD construct was found to have a contaminating band that could not be removed (Figure 4B). The TADH construct was fused to MBP rather than GST because of solubility issues. However, the full-length MBP-E2 fusion protein was equally efficient at stimulating pol ε as the GST-E2 fusion protein (Figure 4C, compare lanes 4–6 with 8–10); accordingly, the use of two different affinity tags was not a confounding issue. These purified recombinant E2 proteins were then evaluated for their ability to stimulate pol ε in M13 primer extension assays (Figure 4D). As expected, the GST and MBP protein domains alone did not result in stimulation of pol ε (Figure 4D, lanes 38–40 and Figure 4E). Full length GST-E2 and the MBP-TADH protein (a deletion of the DBD) both resulted in robust stimulation by pol ε (Figure 4D, lanes 8–10 and 14–16, respectively); however, the TADH was less efficient at stimulating pol ε than the full-length E2 protein. However, these differences were not found to be statistically significant. GST-TAD alone weakly stimulated pol ε (Figure 4D, lanes 11–13); however, GST-Hinge alone resulted in no stimulation of pol ε (Figure 4D, lanes 18–20). All other truncations, which did not contain either the hinge nor the TAD, also showed no detectable stimulation of pol ε (Figure 4D, lanes 17–28). (As noted above, any differences in banding pattern are known to be due to the secondary structure of the bacteriophage DNA template. Variations in pattern between different polymerase preparations and co-factor additions are still not well understood but have been hypothesized to be a result of slight variations in reaction temperature or ionic strength.) This data, along with multiple additional experiments, were quantified using phosphorimage quantification (setting stimulation of pol ε by full-length E2 to 100% in each experiment) and showed that, other than full-length E2, the TAD paired with the hinge domain was the only construct that stimulated pol ε to a similar degree as full length E2 (Figure 4E). This result was found to be statistically significant with a *p* value less than 0.0001 (see Methods and Figure 4 legend). This indicates that the TAD and hinge domains together are important for pol ε stimulation; alone is insufficient. However, there was consistently measurable stimulation of pol ε by TAD alone. Although lower than TADH or E2 FL, stimulation by TAD alone was found to be statistically significant when compared to control lanes (Figure 4E). The E2 DBD appears to be completely dispensable for pol ε stimulation.

## 4. Discussion

E2 plays a critical role in the initiation of PV DNA replication and exhibits other, likely important, interactions with cellular DNA replication proteins. To date, the complete role of E2 in PV DNA replication remains not entirely understood. E2 is mostly considered to be a transcriptional activator and DNA replication initiator protein which helps E1 bind to the PV origin [8]; however, a role for E2 at the PV DNA replication fork during elongation has been suggested [18]. Because we have identified that pol ε interacts functionally with PV E1, we also investigated whether E2 could modulate the activity of pol ε.

In this paper, we have demonstrated a novel functional interaction between PV E2 and cellular pol ε. E2 was observed to stimulate the DNA polymerase activity of pol ε, independently of DNA replication cofactors RFC, PCNA, and RPA (Figure 1). The stimulation of pol ε by E2 did not lead to generation of particularly long or full-length DNA products, such as those observed with RF or E1 [20]. The products generated in the presence of E2 were shorter and the products became even shorter as E2 concentration increased. This is particularly evident in Figure 2 and Figure 4 (Figure 4C, lanes 8–10). The action of E2 did not appear to act through increasing the processivity of pol ε, as addition of increasing levels of template to fixed levels of E2 and pol ε resulted in the synthesis of shorter DNA products (Figure 3). This is consistent with E2 playing an early role in pol ε synthesis, such as in primer acquisition. However, because the primed M13 template lacked E2 binding sites and the E2 DBD was dispensable for this stimulation, this suggests that E2 physically interacts with pol ε to facilitate DNA synthesis, possibly by binding and inducing a conformational change that enhances affinity of pol ε for DNA or the stability of pol ε on the DNA. To date, we have been unable to detect a physical interaction between E2 and pol ε, which suggests that, if there is a physical interaction, it would be transient. This is not uncommon with DNA replication proteins, as they are often involved in transient interactions that “hand off” one protein partner to the next; it often requires very specific reaction conditions and even cross-linking to detect some of these interactions. Although we have not yet detected a E2-pol ε physical interaction, this did not prevent us from identifying this novel functional interaction between E2 and pol ε and merely suggests that we have not yet found the conditions necessary for detection of the physical interaction.

Because E2 has three distinct domains, we analyzed which of these domains was important for the interaction with pol ε. By creating various combinations of the three E2 domains as fusion proteins and testing them for stimulation of pol ε, it was found that the DBD is the only domain of E2 that is fully dispensable for pol ε stimulation; the hinge and TAD domains are both important for stimulation (Figure 4). It has been demonstrated that the functions of E2 are separable, most obviously between the DNA binding (DBD) and the transcriptional transactivation (TAD) [8,17]. Winokur and McBride (17) demonstrated that, in some instances, the DNA binding function of E2 is not required for HPV DNA replication. Although this might, to some degree, be attributable to the important interaction between the E2 TAD and E1, they also found that deletions of or truncations into the hinge domain were also deficient in viral DNA replication, a finding which remains not fully explained [17]. Although we observed stimulation of pol ε with the TAD alone, albeit weak, the presence of the hinge in combination with TAD resulted in robust DNA synthesis by pol ε, nearly equivalent to that of full-length E2. Because the DBD of E2 does not appear to be required for pol ε stimulation, this suggests that the stimulation of pol ε by E2 is not through the DNA-binding function of the E2 DBD targeting pol ε to primer template junctions to increase polymerase cycling. One could speculate that when the TADH domains of E2 interact with pol ε, this could cause a conformational change in pol ε, resulting in either increased affinity for or partial stabilization of pol ε on the DNA. It is possible that the requirement for the hinge domain for pol ε stimulation might be a contributing factor for the previously noted role for the hinge domain in PV DNA replication [17]. That being said, because the sequence of the hinge region is not well-conserved, it is possible that the requirement for the hinge region for full stimulation of pol ε by the TAD might result from a general need for any polypeptide sequence carboxy-terminal to the TAD. Future analyses will investigate the sequence and length requirement for the hinge region for this stimulation.

We have only recently implicated pol ε in PV DNA replication, and the interactions described here are new and remain relatively unexplored. The results shown here suggest that E2 may be playing a role during the initiation of PV DNA replication, possibly assisting pol ε in primer acquisition. As the polymerase departs from the PV origin, it needs to associate with its processivity factors, PCNA and E1, possibly through some sort of “hand-off” mechanism. Any potential competition between binding of pol ε to E2 versus the processivity factors is presumably not a problem if E2 stays primarily associated with the origin (or is displaced from the replication complex). Although we have previously suggested a possible role for E2 in elongation thanks to its binding and stimulation of topoisomerase I [18], topoisomerase I appears to play several roles in assisting SV40 and PV origin recognition [31,32]. Accordingly, this binding and stimulation of E2 to topoisomerase I could be limited to the origin. More study will be required to clarify this interaction and the mechanism of pol ε stimulation by E2.
